# Case Report: Baló's Concentric Sclerosis-Like Lesion in a Patient With Relapsing-Remitting Multiple Sclerosis Treated With Dimethyl Fumarate

**DOI:** 10.3389/fneur.2022.891113

**Published:** 2022-05-23

**Authors:** Karolina Kania, Wojciech Ambrosius, Wojciech Kozubski, Alicja Kalinowska

**Affiliations:** ^1^Department of Neurology, Poznan University of Medical Sciences, Poznan, Poland; ^2^Division of Neurochemistry and Neuropathology, Department of Neurology, Poznan University of Medical Sciences, Poznan, Poland

**Keywords:** multiple sclerosis, dimethyl fumarate, Baló concentric sclerosis, MRI, Baló disease

## Abstract

Baló's concentric sclerosis (BCS) is a rare demyelinating disorder characterized by acute or subacute neurological symptoms associated with characteristic lesions of concentric onion skin appearance on MRI images and in pathology. The connection between BCS and classic MS is still a subject of debates. Our report presents a case of a patient who developed a symptomatic Baló-like lesion following several years of classical relapsing-remitting multiple sclerosis treated with dimethyl fumarate.

Baló disease, also known as Baló's concentric sclerosis (BCS), is a rare inflammatory and demyelinating disorder with a characteristic histopathological picture in which annular areas of demyelination alternate with rings of relatively preserved myelin. It is associated with loss of cerebral white matter oligodendrocytes resembling immunopathological pattern III of multiple sclerosis (MS) lesions. However, contrary to MS, the gray matter is spared. This typical pathology is reflected by magnetic resonance (MR) images of concentric lamellar-like zones of hyper- and hypointensities on T2-weighted sequences, corresponding to myelinated and demyelinated layers. The onion bulb (or wood grain) appearance is distinctive on gadolinium-enhanced T1-weighted sequences (1, 2).

BCS often affects young adults and is nearly two times more common in women and in people from East Asia. Patients may experience acute or subacute symptoms depending on the location of the lesion. In some cases, prodromal symptoms such as malaise, headache, and mild fever were reported. The most prominent features are behavioral changes, headache, muteness, cognitive deficits, aphasia, urinary incontinence, seizures, and hemiparesis that resemble those of intracerebral mass lesions (3). A fulminant disease course, resulting in death, is not uncommon and has recently been reported as 10%. However, complete recovery has also been described (2).

The overlap between BCS and MS is still not well defined. In one study, MRI images of more than half of the subjects revealed co-existence of Baló-like lesions with typical demyelinating MS lesions (4).

Our report is noteworthy, as it is one of the first that describe a patient who, after several years of typical relapsing-remitting MS treated successfully with dimethyl fumarate (and not other therapies previously associated with tumefactive lesions), developed a severe disease exacerbation associated with a new Baló-like lesion.

A 34-year-old female was admitted to the Department of Neurology, Poznan University of Medical Sciences in Poznan, Poland, with acute onset of left hemiparesis in November 2021. Her MS diagnosis was confirmed in April 2016 and was preceded by three relapses: right upper limb paresis in June 2015, cerebellar syndrome in November 2015, and left optic neuritis in February 2016. Her baseline pretreatment brain MRI in March 2016 revealed multiple supra- and infratentorial demyelinating lesions with one gadolinium-enhancing lesion in the cerebellum.

At that time, her cerebrospinal fluid (CSF) results were positive for type II oligoclonal bands. In November 2016, she was treated with dimethyl fumarate 240 mg twice daily; she remained relapse- and progression-free with a stable annual brain MRI scan.

One month before hospitalization, she noticed a weakness in the left lower limb, but the deficit resolved within 7 days without treatment, so she did not seek medical help.

Moreover, for 2–3 weeks, she reported general malaise and subfebrile temperature (37.7°C) without any upper respiratory tract or urinary tract infection symptoms.

Besides multiple sclerosis, she suffered from migraine with aura.

On admission her neurological examination revealed hemiparesis: left upper limb proximally MRC (Medical Research Council-muscle scale) grade 4, distally MRC grade 3, left lower limb proximally and distally MRC grade 2, with diminished sensation in the left limbs, positive Babinski and Rossolimo signs and exaggerated reflexes and mild spasticity in the left limbs.

The lymphocyte count on admission was 1.19 × 10Λ3/ul (normal range 1.1–4.5 × 10Λ3/ul), while 40 days before hospitalization, it was 1.47 × 10Λ3/ul. The white blood cell (WBC) count on admission was 5.96 × 10Λ3/ul (normal range 3.9–11 x × 10Λ3/ul), and 40 days before hospitalization, it was 3.48 × 10Λ3/ul. The patient did not have lymphopenia; but had transient leukopenia in the past.

The clinical picture of this relapse was atypical, because the patient woke up with a fully developed left hemiparesis, which resembled a stroke onset and necessitated an urgent brain MRI.

The study showed a new Baló-like lesion in the right parietal lobe with a size of 27 mm leukopenia 27 mm and the characteristic “onion bulb” appearance. The lesion was partially enhanced after gadolinium contrast (Figures 1A–C). The remaining demyelinating lesions were comparable in terms of number and size with the previous brain MRI obtained in November 2020: 10 plaques in the right and 12 plaques in the left hemisphere.

**Figure 1:**
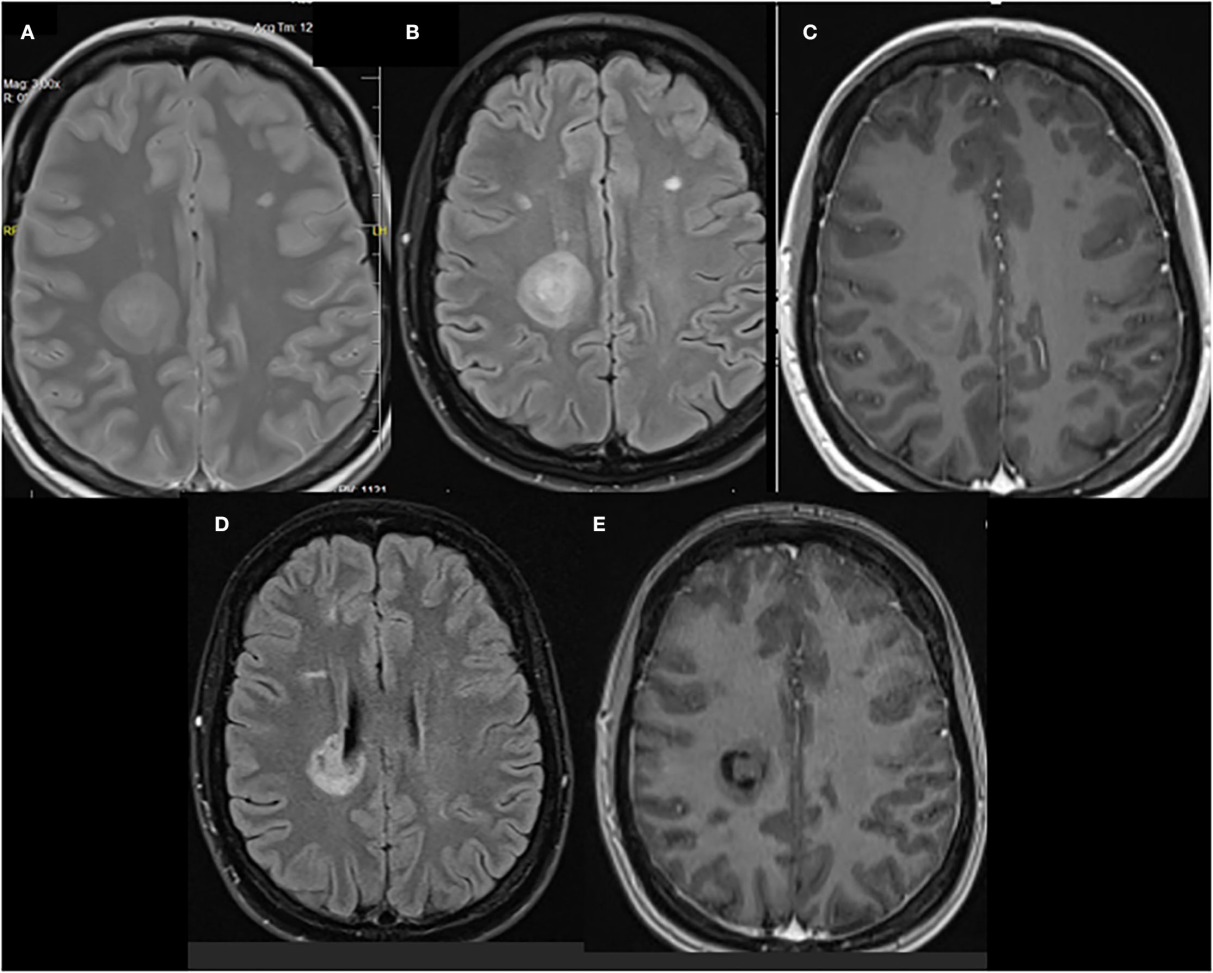
**(A)** Proton density (PD) T2-weighted, **(B)** fluid-attenuated inversion recovery (FLAIR), and **(C)** gadolinium-enhanced T1-weighted images of a right parietal lobe lesion that shows a concentric lamellar appearance that is similar to that of a Balo-like lesion. MRI images 3 months after the treatment were **(D)** FLAIR and **(E)** gadolinium-enhanced T1-weighted.

The patient was treated with methylprednisolone (MPS) 1 g daily for 5 consecutive days without improvement, and the lower left limb deficit progressed to 1 MRC, rendering the patient dependent on a wheelchair.

Therefore, therapeutic plasma exchange (TPE) was implemented. However, the patient suddenly developed a significant clinical deterioration with fever (40°C), shock signs (blood pressure was 70/40 mmHg, tachycardia 150/min) and high inflammation parameters: CRP 156.6 mg/l (on admission 0.5 mg/L, normal range < 5 mg/L), white blood count 12.07 × 10^3^/μl (on admission 5.96 × 10^3^/μl, normal range 3.90–11 × 10^3^/μl), and procalcitonin 21.7 ng/ml (on admission: 0.02 ng/ml, normal range < 0.5 ng/ml).

Chest X-ray was normal, and echocardiogram did not reveal endo- or myocarditis; urine test and antinuclear antibodies also remained also within normal values; COVID-19 antigen tests were negative.

Empirical therapy with ceftriaxone was started, and gradual clinical and laboratory improvement was observed.

Further steroid infusions and TPE were postponed because of signs of shock (possibly septic although blood cultures turned out negative), and the patient was discharged from the hospital to a rehabilitation center with moderate left hemiparesis: upper limb MRC grade 4, lower limb MRC grade 3, and Expanded Disability Status Scale (EDSS) 7 points.

In the 3-month follow-up, a substantial neurological improvement was observed. Her EDDS was 4.5, and the right parietal lobe lesion diminished slightly to a 24-mm size on MRI without gadolinium enhancement (refer to Figures 1D,E). Moreover, the lesion lost its “onion bulb” pattern. In the meantime, her therapy was switched to IV ocrelizumab.

It is still unclear whether BCS is a variant of MS, which typically has a type III immunopattern pathology, or a separate demyelinating disease (3). Data regarding BCS are based on individual cases and limited case series, and our report is an addition to this pool. Baló disease is considered a rare, usually monophasic variant of MS. However, Baló lesions have been shown to coexist with typical demyelinating lesions.

Several publications described patients with relapsing-remitting MS that began as BCS. Ayrignac et al. reported six patients with BCS, all of whom fulfilled MS criteria after 7 years of follow-up, with five of the patients positive for CSF oligoclonal bands (5). Pietroboni et al. described three cases with similar history, two of them fulfilled the MS criteria at baseline or in 10-month follow-up, and treatment with natalizumab or fingolimod was initiated (6). Behrens et al. presented 10 patients with BCS, three of them at baseline fulfilled the 2010 MS diagnostic criteria dissemination in space and in time (7).

On the other hand, the opposite scenario is unusual. There is only anecdotal data about patients who developed BCS after years of clinically stable MS. Moore et al. reported a young female whose third relapse was severe and associated with Baló-like lesions and akinetic mutism. She died 8 months later. Her CSF was negative for oligoclonal bands, which are more common in BCS than in classic MS (8). Barun et al. presented a case of a patient with RRMS who was treated with interferon beta 1a and suffered from aphasia related to a BCS lesion (9). Iannucci et al. described a 31-year-old patient who had both Baló- and MS lesions simultaneously (10). Our patient's relapse was not typical for classic MS, resembling rather a BCS course with a prodromal, subfebrile phase and stroke-like onset, which is consistent with a case series report of 17 patients with BCS, half of whom had prodromal symptoms of malaise, headache, and mild fever (1).

To our knowledge, it is one of the initial reports (2) of a patient with RRMS treated with dimethyl fumarate who developed a Baló-like lesion. However, the possible link between this therapy and tumefactive lesion development remains to be clarified.

The question about the appropriate disease-modifying treatment (DMT) modification for patients who develop Baló-like lesions during their DMT is still open. Our knowledge is based on occasional case reports, which have shown that alemtuzumab was not effective, but that cyclophosphamide, ocrelizumab, natalizumab, interferon beta-1a, and glatiramer acetate could be useful (11, 12). On the other hand, we know that the association between tumefactive lesions and fingolimod treatment is well documented (13–16).

Unfortunately, the cerebrospinal fluid examination was not repeated during the described exacerbation in our patient. This was because of combination of patient-related factors. We do realize this is a limitation, and we do acknowledge that lumbar puncture should be performed in such cases as ours, mainly to exclude the possible neuroinfections (especially in cases associated with lymphopenia or treated with DMTs linked to opportunistic infections). Having said that, based on the clinical picture alone, in our patient, neuroinfectious, especially progressive multifocal encephalopathy (PML), were not likely, as the patient improved significantly. Importantly, it has been shown that oligoclonal bands and IgG index in BCS are more similar to findings in neuromyelitis optica spectrum disorders (NMOSD) than in MS, which could suggest a distinct immunological entity (17).

In conclusion, it seems that publishing all new cases of BCS on DMTs is essential to establish any potential link between MS therapy and rare variants of the disease.

## Data Availability Statement

The original contributions presented in the study are included in the article/supplementary material, further inquiries can be directed to the corresponding author/s.

## Ethics Statement

Written informed consent was obtained from the individual(s) for the publication of any potentially identifiable images or data included in this article.

## Author Contributions

KK and WA drafted the manuscript. WK and AK revised the manuscript critically. All authors read and approved the final version of the manuscript and agreed to be accountable for all aspects of the study.

## Conflict of Interest

The authors declare that the research was conducted in the absence of any commercial or financial relationships that could be construed as a potential conflict of interest.

## Publisher's Note

All claims expressed in this article are solely those of the authors and do not necessarily represent those of their affiliated organizations, or those of the publisher, the editors and the reviewers. Any product that may be evaluated in this article, or claim that may be made by its manufacturer, is not guaranteed or endorsed by the publisher.
